# Family presence during pediatric invasive procedures and resuscitation

**DOI:** 10.1016/j.rpped.2015.07.001

**Published:** 2015

**Authors:** Amélia Gorete Reis

**Affiliations:** a Faculdade de Medicina, Universidade de São Paulo (USP), São Paulo, SP, Brazil

The family presence during invasive procedures and resuscitation in children is becoming more common in pediatric practice, although most emergency services in Brazil do not have structured protocols to better guide this conduct. The opinion of health care professionals and family members on this subject has been discussed in the literature.^1^

Studies evaluating family members’ perception have shown positive factors when they witness such interventions. The family has the opportunity to realize the true severity of the disease or trauma and observe that all that was possible was in fact done, in addition to staying together in a situation of stress, increasing the child's comfort and reducing anxiety. There are reports of families who witnessed their children's resuscitation maneuvers and recommend this conduct to others and there are declarations that grief was eased in the cases when the child died.^2^^,^ 3

Studies assessing professionals’ opinions have shown mixed results. Among the reasons given by professionals to disagree with the presence of family members are: loss of emotional control by the family members and interference with the procedures, the professionals’ discomfort, increasing the chance of failure, limitations in the teaching of trainees, and increased risk of legal suits. Such justifications have been questioned, as they are based more on assumptions than on real facts.

On the other hand, other studies have shown that there are professionals who prefer family involvement. Among the reasons for this preference is the opportunity to educate families about the patient's condition, pressing professionals to consider dignity and privacy when caring for the child, as well as better control of pain and decrease in suffering.^4^^-^ 6

The study by Mekitarian and Angelo,^7^ published in this issue, brings a valuable contribution by assessing the health professionals’ opinion on the family presence in the pediatric emergency room. In addition to being a pioneer study in the national literature, it demonstrates a methodology consistent with high scientific stringency. Mekitarian and Angelo found that younger professionals have better acceptance of family presence during invasive procedures. This fact should not be surprising, as the routine of considering the family as an active participant in the choice of treatment in any situation is a recent one. The discussion about the autonomy of patients and families when facing therapeutic options was introduced at the undergraduate level in health sciences and medical residency and specialization curricula in recent years. Professionals with more seniority (longer time since graduation) were taught to make centralized, arbitrary decisions.^7^

The observation, according to Mekitarian and Angelo,^7^ that the medical team was more favorable than the nursing staff to the family presence during invasive procedures is probably related to these professionals’ practices, that is, more invasive procedures are in general performed by physicians, whereas the less complex ones are performed by the nursing staff.

The results of Mekitarian and Angelo greatly contribute to the creation of training and continuing education strategies for professionals working in emergency rooms in Brazil. It is worth mentioning, however, that care should be exercised in generalizing the results, since the study was carried out in the emergency room of a teaching hospital, where it is expected that professionals be up-to-date and qualified to perform invasive procedures and resuscitation maneuvers.

Many international medical societies have recommended that families be offered the option of staying next to the child during invasive procedures and resuscitation; although Brazil is following this trend, any radicalism to force the adoption of this attitude by all professionals should be avoided, as well as condemning family members who, for several reasons, prefer not to be present. There shall be no impositions that may compromise treatment itself.^8^

The implementation of treatment protocols that include the option of family presence during invasive procedures and emergency treatments should contribute to improving treatment in general in emergency rooms, since it will bring more transparency to therapeutic conducts.^9^
