# Supplementary material for: Family presence during pediatric invasive procedures and resuscitation
Source: Rev Paul Pediatr. 2015 Oct-Dec;33(4):377–8. doi: 10.1016/j.rpped.2015.07.001 (PMC4685554; doi:10.1016/j.rpped.2015.07.001)
Supplement: Translation

# Translation: A presença da família durante procedimentos invasivos e de ressuscitação em pediatria

**Authors:** Amélia Gorete Reis

**Affiliations:** a Faculdade de Medicina, Universidade de São Paulo (USP), São Paulo, SP, Brasil

A presença da família durante procedimentos invasivos e de ressuscitação em crianças vem se tornando mais comum na prática pediátrica, embora a maioria dos serviços de emergência no Brasil não tenha protocolos estruturados que norteiem essa conduta. A opinião de profissionais e de parentes sobre esse assunto vem sendo discutida na literatura.^1^

Estudos que avaliam a percepção dos parentes têm demonstrado fatores positivos quando presenciam tais intervenções. A família tem a oportunidade de perceber a real gravidade da doença ou do trauma e observar que foi realmente feito tudo que era possível, além de manter-se unida numa situação de estresse, o que aumenta o conforto e reduz a ansiedade da criança. Há relatos de famílias que presenciaram a ressuscitação de seus filhos e recomendaram essa conduta às outras e há depoimentos de que o luto foi facilitado em casos de morte da criança.^2^^,^ 3

Estudos que avaliam a opinião dos profissionais apresentam resultados diversos. Dentre os motivos alegados pelos profissionais para discordarem da presença dos parentes estão a perda de controle emocional da família e o prejuízo na execução dos procedimentos, o desconforto dos profissionais, que aumenta a chance de insucesso, a limitação no ensino de profissionais em treinamento e o aumento do risco de processo legal. Tais justificativas vêm sendo questionadas, já que estão baseadas mais em suposições do que em fatos.

Por outro lado, outros estudos demonstram que há profissionais que preferem a participação da família. Dentre as razões que justificam essa conduta, destaca-se a oportunidade de educar as famílias sobre a condição do paciente, forçar os profissionais a pensarem na dignidade e privacidade no cuidado da criança, assim como no melhor controle da dor e redução do sofrimento.^4^^-^ 6

O estudo de Mekitarian e Angelo,^7^ publicado nesta edição, traz contribuição valiosa ao avaliar a opinião dos profissionais de saúde sobre a presença da família na sala de emergência pediátrica. Além de se tratar de estudo pioneiro na literatura nacional, apresenta metodologia consistente com elevado rigor científico. Mekitarian e Angelos observaram que profissionais mais jovens têm melhor aceitação da presença da família durante procedimentos invasivos. Esse fato não deve causar estranheza, já que o hábito de considerar a família como participante ativo na escolha do tratamento frente a qualquer situação é recente. A discussão a respeito da autonomia dos pacientes e parentes frente às opções terapêuticas foi introduzida na graduação nas ciências em saúde e nos currículos de residência e especialização há poucos anos. Profissionais com maior tempo de formação foram ensinados a tomar decisões centralizadas e arbitrárias.^7^

A observação, de acordo com Mekitarian e Angelo,^7^ de que a equipe médica foi mais favorável do que a equipe de enfermagem à presença da família durante os procedimentos mais invasivos provavelmente está relacionada à própria prática desses profissionais, ou seja, procedimentos mais invasivos em geral são do campo de atuação do médico e os menos complexos fazem parte dos cuidados de enfermagem.

Os resultados de Mekitarian e Angelo contribuem sobremaneira para a formulação de estratégias de treinamento e educação continuada de profissionais que atuam em emergências no Brasil. É importante ressaltar, entretanto, que deve haver cuidado na generalização dos resultados obtidos, já que o estudo foi feito em um pronto-socorro de um hospital universitário, onde se espera que os profissionais estejam atualizados e habilitados para a execução de procedimentos invasivos e de ressuscitação.

Muitas sociedades médicas internacionais têm recomendado que se ofereça à família a opção de permanecer ao lado da criança durante procedimentos invasivos e de ressuscitação. Embora a tendência no Brasil caminhe na mesma direção, deve-se evitar o radicalismo de forçar a adoção dessa atitude por todo profissional e/ou condenar parentes que, por motivos diversos, prefiram não estar presentes. De forma alguma deve haver imposição que possa comprometer o tratamento em si.^8^

A implantação de protocolos de atendimento que incluam a opção da presença da família durante procedimentos invasivos e tratamentos de emergência deve contribuir para a melhoria do tratamento de forma global nos prontos-socorros, já que trará mais transparência às condutas terapêuticas.^9^
